# The curious transference of sensations in the ‘mismatched-palm’ rubber hand illusion

**DOI:** 10.1177/20416695251335161

**Published:** 2025-05-11

**Authors:** Nicholas Christos, Jen Mulholland, Margaret O’Leary, Rebekah C. White

**Affiliations:** 1 Pembroke College, University of Oxford, UK; 2 1810 Tufts University, United States; 3 1770 Illinois Wesleyan University, United States; 4 61487 University of Oxford, UK

**Keywords:** body perception, haptics/touch, perception, somatosensory, visuo-haptic interactions

## Abstract

We describe a disconcerting illusion. The participant looks at the *palm* of a *left* rubber hand being touched while receiving synchronous touch on the *back* of their own hidden *right* hand. Despite postural incongruence, mismatching handedness and touch being at a different location on the viewed and hidden hands, participants experience the illusion of ownership of the rubber hand and the illusion of feeling touch on the rubber hand. The robustness of the rubber hand illusion to seemingly profound incongruencies is explained with reference to Riemer et al.’s four basic principles for successful embodiment.

## How to cite this article

Christos, N., Mulholland, J., O'Leary, M., & White, R.C. (2025). The curious transference of sensations in the ‘mismatched-palm’ rubber hand illusion. *i–Perception, 16*(0), 1–4. https://doi.org/10.1177/20416695251335161

In the 25 years since it was first described by Botvinick and Cohen (1998), the rubber hand paradigm has become the flagstone method for eliciting a visuotactile illusion of body ownership. In its most simple form, a *left* rubber hand^1^ is placed – palm facing down – on the table in front of the participant. The participant's own left hand is also placed – palm facing down – on the table, but hidden from view. The experimenter administers synchronous (same timing) touch to both hands. Most participants experience a compelling illusion: it seems that the viewed rubber hand is the participant's hand (ownership) and that the participant is feeling touch *on* the rubber hand (visual capture of touch).

The illusion is explained with reference to multisensory perceptual correlations (see Ehrsson et al., 2004); when what the participant sees on the rubber hand and feels on their own hand are correlated, they interpret the seen and felt inputs as being part of a single event – *vision ‘captures’ touch* and the viewed rubber hand is experienced as if it is the participant's own hand.

Previous studies have shown that, for the illusion to occur, it is important that the participant views touch on a realistic-looking hand rather than an object (Gentile et al., 2013). The viewed hand should be corresponding handedness and posturally-aligned with the participant's hidden hand (Tsakiris & Haggard, 2005). It should also be touched in the corresponding location (Costantini & Haggard, 2007; Riemer et al., 2014).

**Figure 1.**
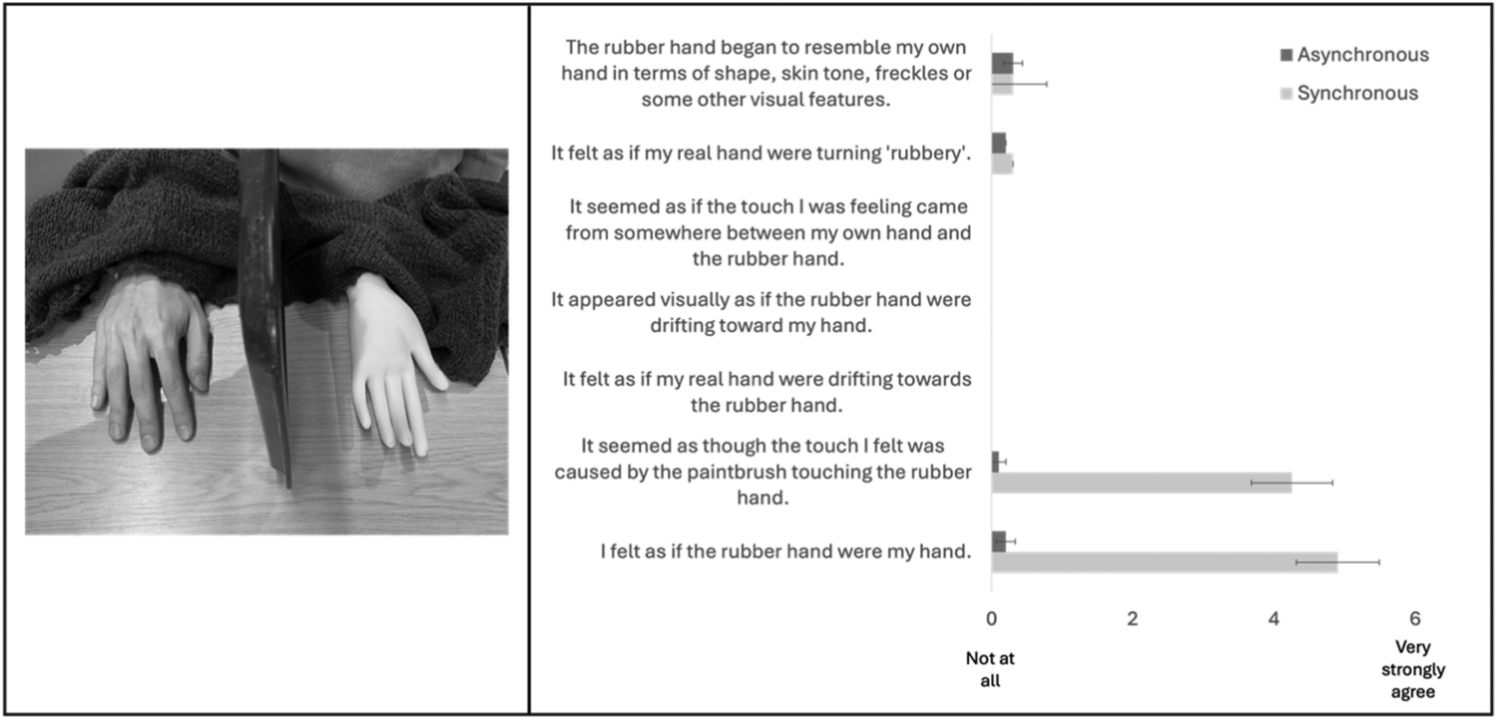
(Left panel) paradigm set–up. Viewed left rubber hand positioned palm facing up with the participant's right hand positioned palm facing down; and vision of the participant's right hand precluded by way of a visual divider – in this case a lunch tray from the College Hall! (Right panel) results of rubber hand questionnaire administered following 120–s synchronous and 120–s asynchronous stimulation (order randomised). The top five questions – as presented on the figure – are control questions and the bottom two questions capture experience of the illusion (order of presentation was randomised). Error bars represent 1 SEM.

In an undergraduate tutorial, we experimented with the set-up of the rubber hand paradigm and stumbled upon a curious variant! A *left* rubber hand was placed *palm facing up* on the table and the participant's own *right* hand was placed *palm facing down* (Figure 1, left panel).^2^ The experimenter administered synchronous strokes and taps to the two hands – ensuring the same finger on each hand was being stroked or tapped, even though for the viewed hand it was on the palm side and for the hidden hand it was the dorsal side. We expected that mismatching handedness, hand orientation and location of touch would break the illusion. And yet, we all experienced a rubber hand illusion. Subsequently, we tested ten naive participants (10 female, *M*_age_ = 21). Experience of the rubber hand illusion was measured using a modified version of Botvinick and Cohen's (1998) questionnaire. When touch was synchronous, nine participants reported the illusion of ownership and visual capture (Figure 1, right panel).

Riemer et al. (2014) investigated mapping of viewed and hidden touch. Participants did not experience the rubber hand illusion when the viewed and hidden hand were touched at a different location; for example, the index finger of the viewed hand and the middle finger of the hidden hand. They proposed four basic principles for successful embodiment of a rubber hand: (a) synchronicity of touch, (b) body unity – whereby the viewed hand must be positioned such that it could be connected to the body, (c) matched rudimental bodily shape and (d) *congruent mapping* of viewed and hidden touch.

Our results can be understood with reference to Riemer et al.'s (2014) four basic principles. Although our set-up involved profound incongruencies, the principles of synchronicity and body unity were met. Moreover, despite incongruent handedness and posture, the thumb was leftmost on both hands (from the participant's perspective) and this seems sufficient for meeting the principle of matched rudimental bodily shape. Finally, whilst touch was administered to a different location – palm viewed hand, back hidden hand – the touched finger was the same and the spatial configuration of that finger, relative to the other fingers was matched. When the experimenter moved between touching the thumb and index finger, for example, this involved the same rightward shift on both hands. Thus, we argue that there was sufficiently congruent mapping of viewed and hidden touch.

The results contribute to work exploring the constraints on the rubber hand illusion. We demonstrate that embodiment is possible, even in the face of seemingly large incongruencies – different handedness, different posture, different location of touch. Congruent mapping of viewed and hidden touch has been identified as necessary to embodiment (Riemer et al., 2014). We show that mapping can meet the congruency requirement when the two hands are touched in different locations (palm vs. back), provided the finger that is touched is the same; a nuance rendered possible by our use of mismatching hands and postures!

## Notes

1. For simplicity we describe the procedure as it applies to a left rubber hand, but a right rubber hand elicits an equally vivid illusion (Ocklenburg et al., 2011).

2. Note that in a study by Ionta et al. (2013), participants also viewed a rubber hand positioned palm upwards while their own hidden hand was positioned palm downward. The set-up differed in that the viewed and hidden hands were both right hands, and both hands were stimulated on the palmar surface. (The participant’s hand was elevated to facilitate this.)
